# Exploring the clinical effects of *Andrographis paniculata*-derived compounds, its extract, or derivatives for the treatment of COVID-19: a systematic review and meta-analysis

**DOI:** 10.3389/fphar.2025.1598255

**Published:** 2025-07-31

**Authors:** Peerapong Prabhakornritta, Neti Waranuch, Anjana Fuangchan, Kantapich Srikham, Kansak Boonpattharatthiti, Cindy Barnig, Siwaporn Boonyasuppayakorn, Tasana Pitaksuteepong, Parvapan Bhattarakosol, Brice Moulari, Yann Pellequer, Teerapon Dhippayom

**Affiliations:** 1 Cosmetics and Natural Products Research Center (CosNat), Faculty of Pharmaceutical Sciences, Naresuan University, Phitsanulok, Thailand; 2 Department of Pharmaceutical Technology, Faculty of Pharmaceutical Sciences and Center of Excellence for Innovation in Chemistry, Naresuan University, Phitsanulok, Thailand; 3 Université Marie et Louis Pasteur, EFS, INSERM UMR1098 RIGHT, Besançon, France; 4 The Research Unit of Evidence Synthesis (TRUES), Faculty of Pharmaceutical Sciences, Naresuan University, Phitsanulok, Thailand; 5 Department of Chest Diseases, University Hospital of Besançon, Besançon, France; 6 Center of Excellence in Applied Medical Virology, Department of Microbiology, Faculty of Medicine, Chulalongkorn University, Bangkok, Thailand; 7 Department of Pharmacotherapy, University of Utah College of Pharmacy, Salt Lake City, UT, United States

**Keywords:** andrographis, andrographolide, COVID-19, antivirals, systematic review, meta-analysis

## Abstract

The COVID-19 pandemic created a global health crisis, with limited effective treatments. *Andrographis paniculata* (Burm. f.) Nees (AP), with known anti-inflammatory and antiviral properties, has been explored as adjunctive therapy for COVID-19, but its clinical evidence remains inconclusive. We hypothesized that AP-derived compounds may improve symptoms and inflammatory responses in mild-to-moderate COVID-19. This systematic review and meta-analysis aimed to evaluate the clinical and biological effects of AP-derived compounds, its extract (APE), or its derivatives in patients with mild-to-moderate COVID-19. A systematic search was conducted in PubMed, EMBASE, CENTRAL, and EBSCO Open Dissertations from January 2020 to October 2024. Randomized controlled trials (RCTs) examining the effects of single-herb AP products compared to antivirals or supportive care (SC) in patients with mild-to-moderate COVID-19 were included if they reported clinical recovery, fever or cough resolution, C-reactive protein (CRP), or interleukin-6 (IL-6) levels. Risk of bias (RoB) was assessed using Cochrane RoB 2.0. A random-effects model was used to estimate pooled effects of included trials, expressed as relative risk (RR) and mean difference (MD) with 95% confidence intervals (CIs). Six RCTs involving 660 adults aged 18 to 60 were included. Compared to antivirals or SC, single-herb AP products showed no significant improvements in fever resolution (RR 1.12; 95%CI 0.90 to 1.38; I^2^ = 0.0%) or cough resolution (RR 0.98; 95%CI 0.74 to 1.31; I^2^ = 47.0%). No significant differences were observed in serum CRP (MD -0.04; 95%CI -0.26 to 0.18; I^2^ = 0.0%) and IL-6 levels (MD -0.07; 95%CI -0.17 to 0.03; I^2^ = 0.0%). While some studies not included in the meta-analysis suggested early reductions in CRP and IL-6, the findings were inconsistent. RoB was high for fever resolution but low for biomarkers. Mild adverse events, primarily liver enzyme elevations, resolved without severe complications. Our systematic review and meta-analysis suggest a potential role for AP extract and its derivatives as adjunctive therapy for COVID-19, with trends indicating possible benefits in symptom improvement and inflammation reduction. These findings highlight the need for further research to explore AP as a complementary therapeutic strategy in COVID-19 management.

**Systematic Review Registration:**

https://www.crd.york.ac.uk/PROSPERO/view/CRD42024608858, identifier CRD42024608858.

## 1 Introduction

The coronavirus disease 2019 (COVID-19) pandemic has caused a global health crisis, affecting millions worldwide through both acute illness and long COVID (Wiersinga et al., 2020; World Health organization, 2021; Greenhalgh et al., 2024; Department of Disease Control, 2023). Early in the pandemic, with no established standard treatment, many countries adopted certain antivirals as the primary available option (Srisubat et al., 2023; Department of Disease Control, 2023). Subsequently, alternative phytotherapies gained increasing attention (Al-Kuraishy et al., 2022; Diantini et al., 2023), with *Andrographis paniculata* (Burm. f.) Nees (AP) emerging as a potential adjunctive therapy, particularly in Thailand (Intharuksa et al., 2022; Diantini et al., 2023; Department of Disease Control, 2023).

AP, a prominent medicinal plant in Asia, has been traditionally used to treat various ailments, including fever, cough, infections, and inflammation. Its bioactive constituents, particularly the diterpenoid lactones andrographolide (AG), neoandrographolide, 14-deoxyandrographolide, and 14-deoxy-11,12-didehydroandrographolide, alongside other phytochemicals such as flavonoids and phytosterols, have been identified and studied for their anti-inflammatory, antiviral, and immunomodulatory properties, notably through modulation of inflammatory pathways (e.g., nuclear factor kappa-light-chain-enhancer of activated B cells (NF-κB), cyclooxygenase-2 (COX-2), and inducible nitric oxide synthase (iNOS)), including enhancement of immune responses via lymphocyte proliferation and interleukin-2 expression (Wagner et al., 2015; Hu et al., 2017; Intharuksa et al., 2022; Siridechakorn et al., 2023; Udupa et al., 2025). While AP has been well-documented in treating other conditions such as upper respiratory tract infections (URTI) (Hu et al., 2017), emerging evidence from *in silico* studies suggests that AP bioactive compounds exhibit strong binding affinities to severe acute respiratory syndrome coronavirus 2 (SARS-CoV-2) targets (Hiremath et al., 2021; Udupa et al., 2025), supporting its potential role in COVID-19 management, which remains under investigation (Intharuksa et al., 2022). Commercial AP products are available globally, including single-herb formulations such as Fa Thalai Chon extract tablets or capsules (Thailand), Chuan Xin Lian and Xiyanping (China), and HMPL-004 and Andrographis EP80 (India) (Udupa et al., 2025).

Despite numerous studies evaluating the efficacy of AP in treating COVID-19, significant research gaps remain regarding its effectiveness in the clinical context (Diantini et al., 2023). Although preliminary data suggest potential benefits, the findings have been inconsistent, underscoring the need for further investigation (Al-Kuraishy et al., 2022; Intharuksa et al., 2022; Diantini et al., 2023). Given the global demand for accessible, affordable, and plant-derived therapeutic options against COVID-19, clarifying AP’s clinical value is essential. This systematic review and meta-analysis aimed to aggregate and evaluate the clinical and biological outcomes of AP-derived compounds, APE, or its derivatives as adjunctive therapy for mild-to-moderate COVID-19, addressing existing knowledge gaps and providing a clearer understanding of AP’s potential therapeutic role.

## 2 Methods

This study was conducted in accordance with the methodological standards outlined in the Cochrane Handbook for Systematic Reviews of Interventions (Higgins et al., 2024) and the Preferred Reporting Items for Systematic Reviews and Meta-Analyses (PRISMA) 2020 statement (RRID:SCR_018721) (Page et al., 2021a; Page et al., 2021b; Akl et al., 2024). The protocol was prospectively registered in PROSPERO (RRID:SCR_019061) (CRD42024608858).

### 2.1 Information sources and search strategy

The search strategy was developed based on the established PICO framework and implemented across multiple databases, including PubMed (RRID:SCR_004846), EMBASE (RRID:SCR_001650), Cochrane Central Register of Controlled Trials (CENTRAL) (RRID:SCR_006576), and EBSCO Open Dissertations. A comprehensive search was conducted to identify studies published between 1 January 2020, and 3 October 2024. A combination of free-text keywords and database-specific terms or thesauri was employed for each database, with search strategies encompassing three key domains: (1) COVID, (2) Andrographis, and (3) antivirals. The complete search strategy for each database was detailed in Supplementary Table S1. To further enhance the search, SCOPUS (RRID:SCR_022559) was utilized for the reference tracking (snowball technique), and Google Scholar (RRID:SCR_008878) was employed for forward citation tracking.

### 2.2 Eligibility criteria

This review included randomized controlled trials (RCTs) that met the following eligibility criteria based on the PICO framework: Population—human participants with mild-to-moderate (non-severe) COVID-19 (World Health organization, 2021); Intervention—AP-derived compounds, APE, or its derivatives administered as adjunctive therapy, without co-administration of other herbal extracts; Comparator—antivirals or supportive care (SC) provided for non-severe COVID-19; Outcomes—studies were required to report at least one of the following: overall clinical recovery, fever resolution, cough resolution, or objective laboratory biomarkers such as serum CRP or interleukin-6 (IL-6) levels. Two independent reviewers (PP and KS) screened titles and abstracts, followed by full-text assessment by two reviewers (PP and KS). Disagreements were resolved through consensus or consultation with a third reviewer (KB, AF, or TD).

### 2.3 Data extraction

Data extraction was systematically conducted using a pre-defined form aligned with the Consolidated Standards of Reporting Trials (CONSORT)-Herbal Medicinal Interventions checklist for RCTs (Gagnier et al., 2006a; Gagnier et al., 2006b). Two independent reviewers (PP and KS) extracted data, with disagreements resolved by a third reviewer (KB, AF, or TD). Extracted data included study characteristics (author, year, country, setting, and duration), population demographics (age, sex, and COVID-19 severity), and intervention details (product characteristics, dosage regimens of AP-derived compounds, APE, or its derivatives), and specifics of comparators, including antiviral agents or SC. Efficacy outcomes included clinical recovery, symptom resolution (fever and cough), and serum biomarkers (CRP and IL-6), with their respective time points. Additionally, adverse outcomes, such as hepatic and renal impairments, were documented.

### 2.4 Quality assessment

The quality of the included studies was assessed using the Cochrane Risk of Bias (RoB) 2.0 tool (Sterne et al., 2019). Two reviewers (PP and KS) independently evaluated each study across all domains, categorizing them as either low risk, some concerns, or high risk. The overall risk of bias was determined based on the highest risk level across domains: low if all were low, some concerns if at least one raised concerns but none were high, and high if one or more domains were classified as high risk. In instances of disagreement between the reviewers, discussions were held, and a third reviewer (KB, AF or TD) was consulted to make the final determination. The RoB assessment results were visualized using a traffic light plot generated via the Risk-of-bias VISualization (robvis) web application (RRID:SCR_018755) (McGuinness and Higgins, 2021).

### 2.5 Data synthesis

Data synthesis involved both descriptive and quantitative approaches, depending on data availability and nature. A narrative summary was provided for each outcome, detailing consistent findings, notable differences, and factors that could influence the results, such as variations in study design, population characteristics, or intervention protocols. For outcomes with sufficient data, a pairwise meta-analysis was conducted using a random-effects model (DerSimonian and Laird method) to estimate overall effect sizes (DerSimonian and Laird, 1986). Relative risk (RR) was used to measure the effect on overall clinical recovery, fever and cough resolution, including high level of serum CRP; whereas mean difference (MD) was used to measure the effect on serum CRP and IL-6 levels. These measures of effects were accompanied by their corresponding 95% confidence interval (CIs).

Heterogeneity among studies was assessed using Chi-squared (χ^2^) test, as well as I^2^ statistic, with thresholds for heterogeneity interpreted as follows: 0%–40% might not be important, 30%–60% indicating moderate heterogeneity, 50%–90% indicating substantial heterogeneity, and 75%–100% indicating considerable heterogeneity (Higgins and Thompson, 2002). All statistical analyses were performed using Review Manager (RevMan) version 5.4 (RRID:SCR_003581; legacy version with existing license), with results visually presented as forest plots where applicable (The Cochrane Collaboration, 2020).

## 3 Results

### 3.1 Study selection

**FIGURE 1:**
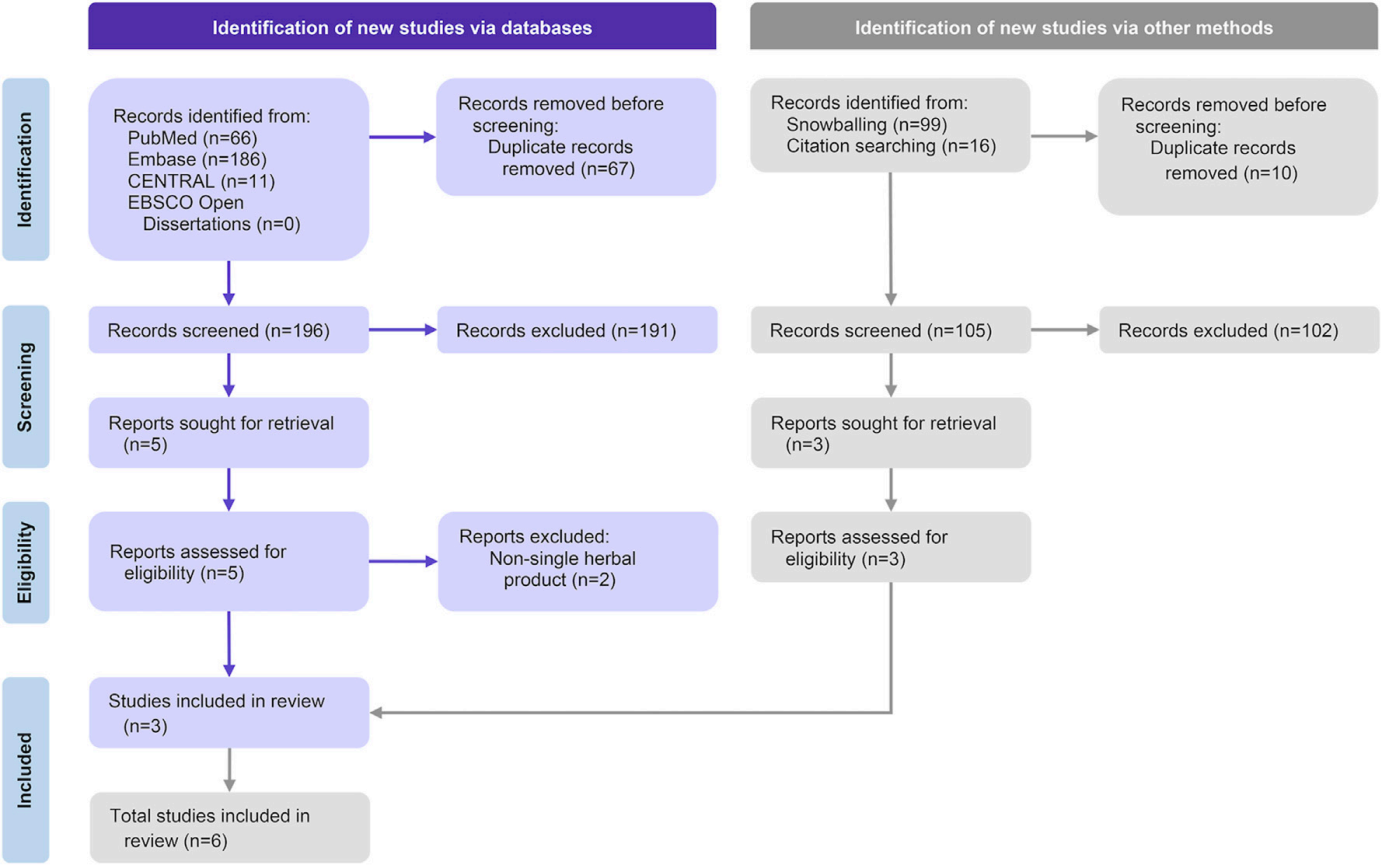
The preferred reporting items for systematic reviews and meta-analyses (PRISMA) flow diagram of selected articles.

A total of 301 articles were identified after removing duplicates, including 196 from electronic databases searches and 105 from snowballing and citation tracking. Six studies, involving 660 participants, were included in the systematic review (Wanaratna et al., 2022; Zhang et al., 2021; Kanokkangsadal et al., 2023; Shanker et al., 2023; Siripongboonsitti et al., 2023; Prasoppokakorn et al., 2024). A meta-analysis was conducted on four outcomes from two of these studies, encompassing 228 participants (Siripongboonsitti et al., 2023; Prasoppokakorn et al., 2024) (Figure 1).

### 3.2 Study characteristics

Participants in the included studies were adults aged 18 to 60 with mild-to-moderate COVID-19, treated with adjunctive AP-derived compounds, APE, or its derivatives. The studies were conducted across multiple regions in Asia, including Thailand (Wanaratna et al., 2022; Kanokkangsadal et al., 2023; Siripongboonsitti et al., 2023; Prasoppokakorn et al., 2024), India (Shanker et al., 2023), and China (Zhang et al., 2021), with various study timeframes from 2020 to 2022. These studies took place in diverse healthcare settings, ranging from state quarantine facilities, field hospitals, university hospitals, and academic institutions, highlighting variations in healthcare practices and resource availability. Sample sizes ranged from 57 to 165, with follow-up periods ranging from 5 to 21 days post-intervention. Academic grants were the most frequently reported funding source, with all studies disclosing their funding details (Table 1).

**TABLE 1:** Characteristics of included studies.

Study, year (country)	Setting	Population (n)	Age (y)	Male (%)	Duration (day)	Experimental intervention	Comparator intervention	Outcome measures	Funding support
Prasoppokakorn, 2024 (Thailand) (Prasoppokakorn et al., 2024)	Multicentred, 2 provincial hospitals	Mild-to-moderate COVID-19 (n = 82)	43.9 ± 15.2	53.66	14	APE (60 mg AG/capsule), 1 capsule po tid (providing 180 mg AG/day); adjunctive to Favipiravir and SC (for 5 days)	Favipiravir (a loading dose of 3,600 mg on day 0, followed by a daily dose of 1,600 mg from days 1–4) with SC	Fever and cough resolution rates, CRP, IL-6, and adverse outcomes (elevated average liver enzymes without hepatitis)	Academic funding
Siripongboonsitti, 2023 (Thailand) (Siripongboonsitti et al., 2023)	Single centred, 1 hospital	Mild and moderate COVID-19 (n = 146)	EG 35 (26–46) CG 41 (28–51)	43.84	14	APE (20 mg AG/capsule), 3 capsules po tid (providing 180 mg AG/day); adjunctive to Favipiravir and SC (for 5 days)	Placebo 3 capsules po tid and favipiravir (a loading dose of 3,600 mg on day 0, followed by a daily dose of 1,600 mg from days 1 to 4) with SC	WHO-CPS improvement, fever and cough resolution rates, CRP, IL-6, and adverse outcomes (mild and moderate hepatitis)	Academic funding
Shanker, 2023 (India) (Shanker et al., 2023)	Multicentred, 2 hospitals	Mild-to-moderate COVID-19 (n = 80)	41–60	68.75	4	CIM-MEG19 (200 mg providing APE 150 mg/tablet, with analyzed AG 35–40 mg/g), 1 tablet po bid; adjunctive to SC (n = 40) (for 4 days)	SC (n = 3) or SC with antivirals (remdesivir (n = 24), favipiravir (n = 13))	Time to WHO ordinal clinical severity scale (2-point) improvement, hs-CRP, IL-6, negative COVID-19 PCR	Healthcare industry funding
Kanokkangsadal, 2023 (Thailand) (Kanokkangsadal et al., 2023)	Single centred, university field hospital	Mild-to-moderate COVID-19 (n = 165)	EG 29 (23, 35) CG 33 (25, 42)	33.33	5	APE (20 mg AG/capsule), 3 capsules po tid (providing 180 mg AG/day); with SC (for 5 days)	Placebo 3 capsules po tid and SC	WHO-CPS improvement (changing >3), Fever and cough severity (clinical COVID-19 symptoms 0–10 numeric rating scale)	Academic funding
Wanaratna, 2022 (Thailand) (Wanaratna et al., 2022)	Multicentred, 2 state quarantine hospitals	Mild COVID-19 (n = 57)	EG 39.3 ± 11.4 CG 39.4 ± 11.6	40.35	5	APE (20 mg AG/capsule), 3 capsules po tid (providing 180 mg AG/day); with SC (for 5 days)	Placebo 3 capsules po tid and SC	Self-assessed complete clinical recovery from VAS scores, low CRP level (≤10 mg/L), negative COVID-19 RT-PCR	Academic funding
Zhang, 2021 (China) (Zhang et al., 2021)	Multicentred, 5 hospitals	Mild-to-moderate COVID-19 (n = 130)	EG 44.31 ± 13.45 CG 48.25 ± 14.22	46.15	14	Xiyanping (9-dehydro-17-hydroandrographolide, and sodium 9-dehydro-17-hydro-andrographolide-19-yl sulfate) injection (10 mg AG/kg, not exceed 500 mg/day); with SC (for 7–14 days)	SC	Time to complete symptom, fever, and cough resolutions, and time to virus clearance (2 consecutive nucleic acid tests)	Academic funding

Data are reported as mean ± standard deviation (SD) or mean (range) or median (first quartile (Q1), third quartile (Q3)).

EG, experimental group; CG, comparator group; COVID-19, coronavirus disease of 2019; HT, hypertension; DM, diabetes; DLP, dyslipidemia; CVD, cardiovascular diseases; CKD, chronic kidney disease; EG, experimental group; CG, comparator group; APE, *Andrographis paniculata* (Burm. f.) nees extract; AG, andrographolide; SC, supportive care; WHO-CPS, COVID-19, World Health Organisation clinical progression scale; VAS, visual analogue scale; CRP, C-reactive protein; hs-CRP, high-sensitivity CRP; IL, interleukin; RT-PCR, real-time polymerase chain reaction; po, per os (oral administration); tid, ter in die (three times a day); od, omne in die (once a day).

The use of APE varied among studies, with five using oral administration of APE for 4 to 5 consecutive days. Of these, four studies administered 180 mg of AG per day (Wanaratna et al., 2022; Kanokkangsadal et al., 2023; Siripongboonsitti et al., 2023; Prasoppokakorn et al., 2024), while one provided 14–16 mg AG per day (Shanker et al., 2023). One study utilized a mixture of two synthetic AG derivatives administered intravenously (IV) as the experimental intervention (Zhang et al., 2021). For ethical reasons, standalone antivirals use without SC was not permitted during the COVID-19 pandemic. Comparators included antivirals [two studies (Siripongboonsitti et al., 2023; Prasoppokakorn et al., 2024)], SC alone [three studies (Wanaratna et al., 2022; Zhang et al., 2021; Kanokkangsadal et al., 2023)], and a combination of antivirals and SC [one study (Shanker et al., 2023)]. SC for COVID-19 primarily involved symptomatic treatment based on national clinical practice guidelines, typically including antipyretics, analgesics, and antitussives (Centers for Disease Control and Prevention, 2024). Among the included studies, five did not provide details regarding SC (Wanaratna et al., 2022; Zhang et al., 2021; Shanker et al., 2023; Siripongboonsitti et al., 2023; Prasoppokakorn et al., 2024), and only one study specified the supportive medications used (i.e., acetaminophen and antihistamines) (Kanokkangsadal et al., 2023).

### 3.3 Quality of included studies

**FIGURE 2:**
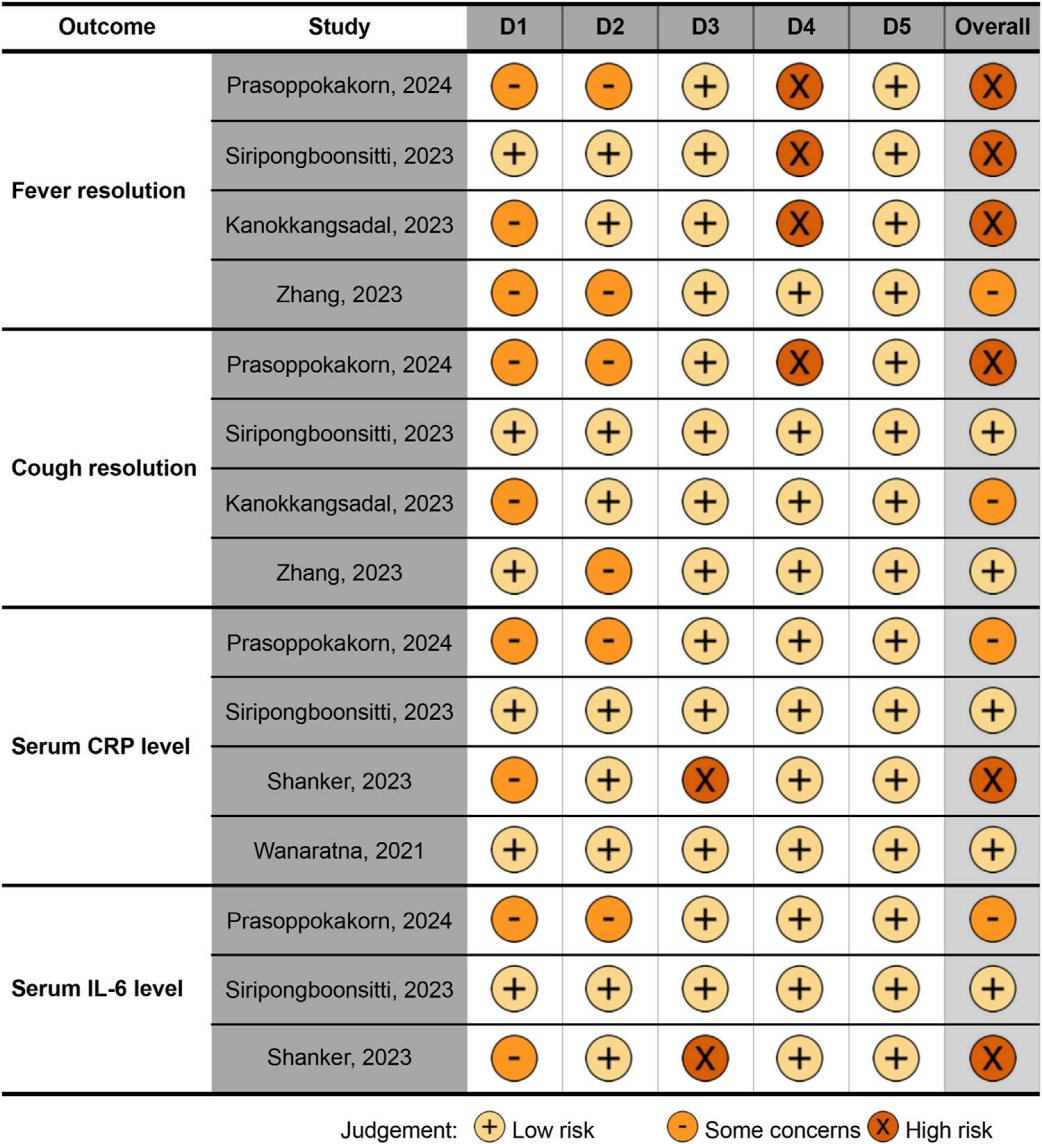
Quality (risk of bias) of the included studies, visualized by the traffic light plot. Domains: D1, bias arising from the randomization process; D2, bias due to deviations from intended intervention; D3, bias due to missing outcome data; D4, bias in measurement of the outcome; D5, bias in selection of the reported result. CRP, C-reactive protein; hs-CRP, high-sensitivity CRP; IL, interleukin.

The Cochrane overall RoB assessment showed variability across the included studies and outcomes. For fever resolution, three studies were rated as having a high RoB (Kanokkangsadal et al., 2023; Siripongboonsitti et al., 2023; Prasoppokakorn et al., 2024), while one study had some concerns (Zhang et al., 2021), highlighting significant methodological limitations. For cough resolution, the RoB varied, with one study at high risk (Prasoppokakorn et al., 2024), one showing some concerns (Kanokkangsadal et al., 2023), and two rated as low risk (Zhang et al., 2021; Siripongboonsitti et al., 2023), reflecting heterogeneity in evidence robustness. For CRP levels, one study had a high RoB (Shanker et al., 2023), one had some concerns (Prasoppokakorn et al., 2024), and two were assessed as low risk (Wanaratna et al., 2022; Siripongboonsitti et al., 2023), suggesting greater reliability for this outcome. Finally, for IL-6 levels, one study had a high RoB (Shanker et al., 2023), one had some concerns (Prasoppokakorn et al., 2024), and one was deemed low risk (Siripongboonsitti et al., 2023), indicating variability in the quality of available evidence (Figure 2).

### 3.4 Effects of *Andrographis paniculata*

**FIGURE 3:**
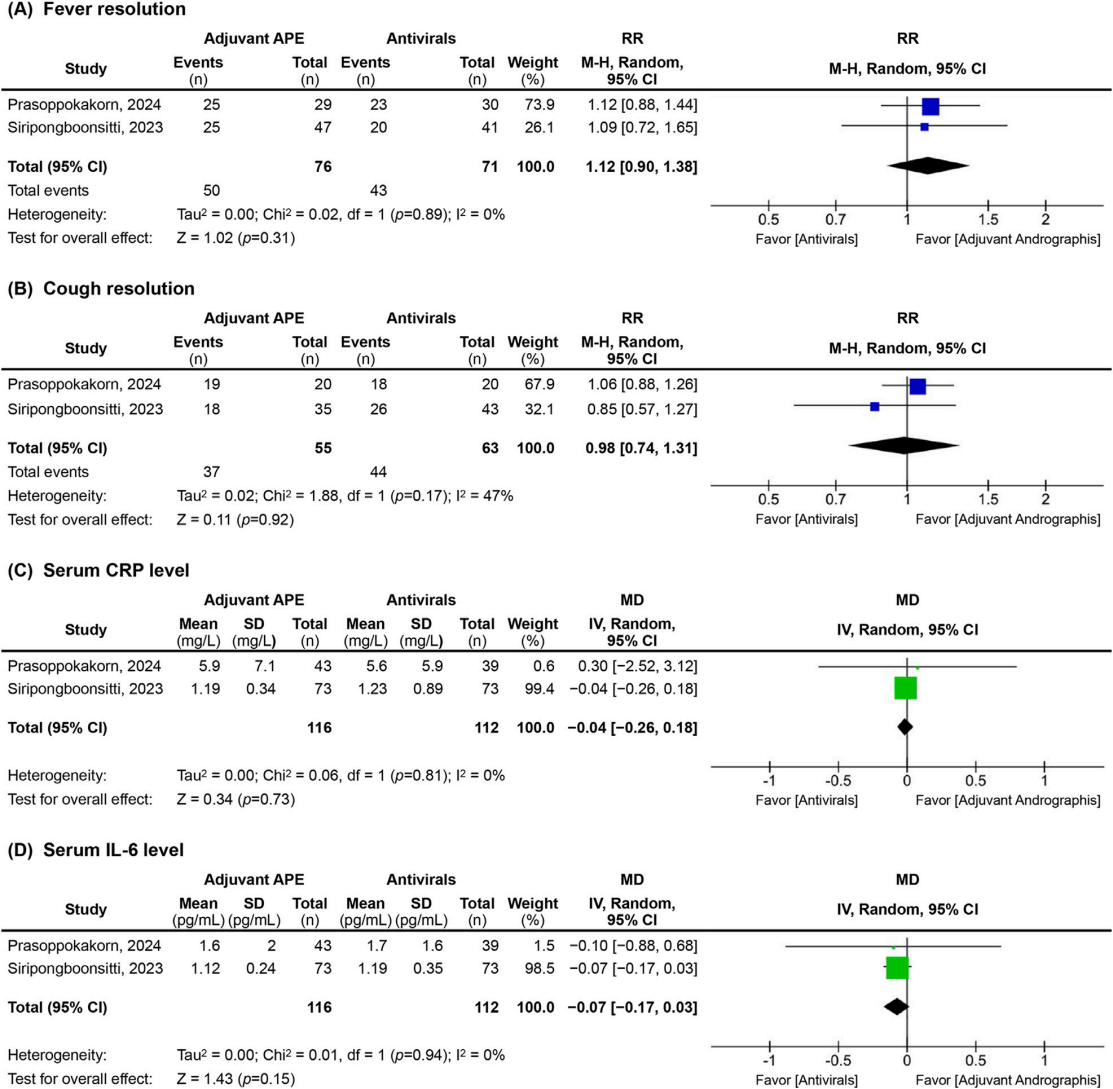
Forest plots of clinical and biological outcomes, including **(A)** fever resolution, **(B)** cough resolution, **(C)** serum C-reactive protein (CRP), and **(D)** interleukin-6 (IL-6) levels, assessed at the final time point of the included studies. RR, relative risk; CI, confidence interval; MD, mean difference; SD, standard deviation; M-H, Mantel-Haenszel method; Random, a random-effects model; IV, intervention; df, degree of freedom; APE, *Andrographis paniculata* extract.

The synthesis of evidence from the six included studies revealed a complex landscape of significant and non-significant findings across the targeted clinical and biomarker outcomes, including fever and cough resolution, as well as serum CRP and IL-6 levels. These outcomes were assessed in diverse clinical contexts and time points. The findings from each included trial were described narratively (Table 2), followed by pooled estimates derived from the meta-analysis (Figure 3), providing a more comprehensive interpretation of the results.

**TABLE 2:** Effects of Andrographis paniculata-derived compounds, its extract, or derivatives on COVID-19.

Outcome	Study, year (country)	Outcome measurement	Day of measurement	Effect in experimental group	Effect in comparator group	*p*-value	References
Adjunctive AP vs. Antivirals							
Clinical recovery	(Siripongboonsitti et al., 2023) (Thailand)	WHO Clinical Progression Scale improvement (≥1 scale improvement)	2	31/72 (43.06)	27/73 (36.99)	0.456^a^	Siripongboonsitti et al. (2023)
			5	41/71 (57.75)	33/71 (46.48)	0.179^a^	
			14	60/70 (85.71)	55/71 (77.46)	0.207^a^	
	(Shanker et al., 2023) (India)	Time to WHO ordinal clinical severity scale (2-point) improvement (day)		After treatment 4.17 ± 0.56 Baseline 3.02 ± 0.30	After treatment 6.23 ± 1.95 Baseline 3.17 ± 0.30	<0.001^e^	Shanker et al. (2023)
Fever resolution	(Siripongboonsitti et al., 2023) (Thailand)	Fever resolution (VAS at 0 point)	4	18/35 (51.43)	26/43 (60.47)	0.423^a^	Siripongboonsitti et al. (2023)
		Fever clinical improvement (>50% improvement)	4	26/35 (74.29)	32/43 (74.42)	0.989^a^	
	(Prasoppokakorn et al., 2024) (Thailand)	Fever resolution	7	10/20 (50.00)	9/20 (45.00)	0.608^b^	Prasoppokakorn et al. (2024)
			14	19/20 (95.00)	18/20 (90.00)	0.602^b^	
Cough resolution	(Siripongboonsitti et al., 2023 (Thailand)	Cough resolution (VAS at 0 point)	4	25/47 (53.19)	20/41 (48.78)	0.680^a^	Siripongboonsitti et al. (2023)
		Cough clinical improvement (>50% improvement)	4	34/47 (72.34)	25/41 (60.98)	0.258^a^	
	(Prasoppokakorn et al., 2024) (Thailand)	Cough resolution	7	16/29 (55.17)	8/30 (26.67)	0.017^b^	Prasoppokakorn et al. (2024)
			14	25/29 (86.21)	23/30 (76.67)	0.025^b^	Siripongboonsitti et al. (2023)
Serum CRP level	(Siripongboonsitti et al., 2023) (Thailand)	Serum hs-CRP level (mg/L)	2	4.81 (2.21–8.82)	3.08 (1.55–7.47)	0.103^d^	
			5	2.82 (1.28–6.28)	3.17 (1.13–9.03)	0.694^d^	
			14	1.19 (0.59–1.93)	1.23 (0.56–4.03)	0.333^d^	
	(Prasoppokakorn et al., 2024) (Thailand)	Serum CRP level (mg/L)	7	5.8 ± 7.3	18.4 ± 31.4	0.019^c^	Prasoppokakorn et al. (2024)
			14	5.9 ± 7.1	5.6 ± 5.9	0.857^c^	
Serum IL-6 level	(Siripongboonsitti et al., 2023) (Thailand)	Serum IL-6 level (pg/mL)	5	1.61 (0.86–3.56)	1.98 (1.05–4.47)	0.277^d^	Siripongboonsitti et al. (2023)
			14	1.12 (0.72–1.65)	1.19 (0.78–2.15)	0.339^d^	
	(Prasoppokakorn et al., 2024) (Thailand)	Serum IL-6 level (pg/mL)	7	2.0 ± 2.4	21.8 ± 68.3	0.001^c^	Prasoppokakorn et al. (2024)
			14	1.6 ± 2.0	1.7 ± 1.6	0.792^c^	
Adjunctive AP vs. Supportive care alone							
Clinical recovery	(Kanokkangsadal et al., 2023) (Thailand)	WHO-CPS improvement (>3 scales improvement)	5	79/83 (95.18)	74/82 (90.24)	0.222^a^	Kanokkangsadal et al. (2023)
	(Wanaratna et al., 2022) (Thailand)	Complete clinical recovery from self-assessed VAS scores	5	0/29 (0.00)	0/28 (0.00)		Wanaratna et al. (2022)
	(Zhang et al., 2021) (China)	Time to complete symptom resolution (day)		8.33 ± 4.87	11.86 ± 6.93	0.008^f^	Zhang et al. (2021)
Fever resolution	(Kanokkangsadal et al., 2023) (Thailand)	Fever severity from clinical COVID-19 symptoms (0–10 numeric rating scale)	2	−1 (−3, 0)	0 (−2.25, 0)	0.210^d^	Kanokkangsadal et al. (2023)
			3	−1 (−4, 0)	0 (−3, 0)	0.153^d^	
			4	−1 (−4, 0)	0 (−3.25, 0)	0.336^d^	
			5	−1 (−4, 0)	0 (−4, 0)	0.546^d^	
	(Zhang et al., 2021) (China)	Time to fever resolution (day)		3.33 ± 2.76	4.60 ± 3.55	0.075^f^	Zhang et al. (2021)
Cough resolution	(Kanokkangsadal et al., 2023) (Thailand)	Cough severity from clinical COVID-19 symptoms (0–10 numeric rating scale)	2	−1 (−2, 0)	−1 (−3, 0)	0.448^d^	Kanokkangsadal et al. (2023)
			3	−1 (−2, 0)	−1 (−3, 0)	0.448^d^	
			4	−2 (−3, 0)	−2 (−3, 0)	0.98^d^	
			5	−2 (−3, −1)	−2 (−3.25, 0)	0.836^d^	
		Cough frequency from clinical COVID-19 symptoms (0–10 numeric rating scale)	2	−1 (−2, 0)	−1 (−2, 0)	0.654^d^	
			3	−1 (−3, 0)	−1 (−3, 0)	0.793^d^	
			4	−1 (−3, −1)	−1 (−3, 0)	0.862^d^	
			5	−2 (−3, −1)	−1 (−3, 0)	0.919^d^	
	(Zhang et al., 2021) (China)	Time to cough resolution (day)		6.89 ± 4.33	12.25 ± 6.85	0.001^f^	Zhang et al. (2021)
Serum CRP level	(Shanker et al., 2023) (India)	Serum CRP level (mg/L)	4	After treatment 9.98 ± 14.5 Baseline 21.5 ± 41.5	Not reported	0.01^7^ ^h^	Shanker et al. (2023)
	(Wanaratna et al., 2022) (Thailand)	Low serum CRP level (≤10 mg/L)	5	29/29 (100.00)	23/28 (82.14)	0.023^a^	Wanaratna et al. (2022)
Serum IL-6 level	(Shanker et al., 2023) (India)	Serum IL-6 level (pg/mL)	4	After treatment 8.69 ± 6.35 Baseline 7.13 ± 2.33	Not reported	0.01^7^ ^h^	Shanker et al. (2023)

Effects in each groups are reported as mean ± standard deviation (SD) or mean (range) or median (first quartile (Q1), third quartile (Q3)) or differences in median (first quartile (Q1), third quartile (Q3)).; HR, hazard ratio; RR, risk ratio.

COVID-19, coronavirus disease of 2019; EG, experimental group; CG, comparator group; AP, *Andrographis paniculata* (Burm. f.) nees; APE, AP, extract; SC, supportive care; WHO-CPS, COVID-19, World Health Organization clinical progression scale; VAS, visual analogue scale; CRP, C-reactive protein; hs-CRP, high-sensitivity CRP; IL, interleukin; RT-PCR, real-time polymerase chain reaction; NA, nucleic acid.

^a^ Chi-square test.

^b^ Fisher’s exact test.

^c^ Independent t-test.

^d^ Mann-Whitney U test.

^e^ Levene’s test for equality of variances.

^f^ Log-rank test with Cox proportional hazard model.

^g^ F test.

^h^ comparing to baseline.

#### 3.4.1 Overall clinical recovery

Overall clinical recovery was defined by either improvement on the WHO Clinical Progression Scale (WHO-CPS) (≥1 (Siripongboonsitti et al., 2023) or >3 (Kanokkangsadal et al., 2023) points), a 2-point improvement on the WHO ordinal clinical severity scale (WHO-CSS) (Shanker et al., 2023), complete recovery based on self-assessed visual analog scale (VAS) scores (Wanaratna et al., 2022), or complete symptom resolution (Zhang et al., 2021). The efficacy of AP on overall clinical recovery varied across the included studies. While three studies (Wanaratna et al., 2022; Kanokkangsadal et al., 2023; Siripongboonsitti et al., 2023) found no significant difference between AP and comparators at 2 (Siripongboonsitti et al., 2023), 5 (Wanaratna et al., 2022; Siripongboonsitti et al., 2023), and 14 (Siripongboonsitti et al., 2023) days, Shanker’s 2023 study reported a significantly shorter time to a 2-point improvement on the WHO-CSS (*p* < 0.001) with AP compared to the comparator (mostly antivirals) (Shanker et al., 2023). Similarly, Zhang’s 2021 study demonstrated a significantly shorter time to complete symptom resolution in the AP group compared to the SC group (*p* = 0.008) (Zhang et al., 2021).

#### 3.4.2 Fever resolution

Fever resolution, which was defined by the absence of symptoms recorded by the research team or a 0 scale (‘I have no fever at all’) from the self-assessed VAS, was not significantly different between groups across all included studies (Table 2; Figure 3a). While Zhang’s 2021 study reported a trend towards faster fever resolution in the AP group over the SC group (Zhang et al., 2021), this finding did not reach statistical significance.

Our meta-analysis from two trials comparing AP with antivirals (n = 147) (Siripongboonsitti et al., 2023; Prasoppokakorn et al., 2024) confirmed this finding, with a RR at the end of follow-up of 1.12 (95%CI; 0.90–1.38) with no heterogeneity (I^2^ = 0.0%).

Based on the available evidence, there is insufficient support to conclude that AP-derived compounds, APE, or its derivatives, are effective in reducing fever in non-severe COVID-19 patients.

#### 3.4.3 Cough resolution

Cough resolution was defined as the absence of symptoms recorded by the research team or a score of 0 (‘I have no cough at all’) evaluated by a self-assessed VAS at indicated time points. The findings for cough resolution were mixed. The pooled estimates, based on two trials (n = 118) (Siripongboonsitti et al., 2023; Prasoppokakorn et al., 2024), demonstrated the RR of cough resolution in the final follow-up of 14 days for AP compared to antivirals was 0.98 (95% CI; 0.74 to 1.31; I^2^ = 47.0%) (Figure 3b).

On the other hand, individual studies suggested significant short-term advantages. For instance, Prasoppokakorn’s 2024 study reported a statistically significant improvement in cough resolution rates at both 7 days (*p* = 0.017) and 14 days (*p* = 0.025) in the APE group compared to the antiviral group (Prasoppokakorn et al., 2024). Moreover, Zhang’s 2021 study demonstrated that the AP group recovered from coughs nearly twice as fast as the SC group (*p* = 0.001) (Zhang et al., 2021) (Table 2). These results indicated a potential time-sensitive effect of APE, implying that its therapeutic impact may be more pronounced during the early stages of illness.

#### 3.4.4 Serum CRP levels

The analysis of inflammatory biomarkers, particularly serum CRP levels, revealed variation in the observed effects, potentially linked to the timing of assessments. Comparing AP with antivirals in two trials (n = 228) (Siripongboonsitti et al., 2023; Prasoppokakorn et al., 2024) the pooled estimates did not show a significant reduction in CRP levels between groups by the final follow-up, with an overall MD of −0.04 (95%CI; −0.26 to 0.18) with no heterogeneity (I^2^ = 0.0%) (Figure 3c).

However, earlier measurements demonstrated noteworthy results. In the study by Prasoppokakorn, serum CRP levels in the APE group were significantly lower at 7 days compared to the antiviral group (*p* = 0.019), although this difference was no longer evident at 14 days (Prasoppokakorn et al., 2024). Additionally, Wanaratna’s 2022 study reported that, by day 5, a greater proportion of patients in the APE group achieved low serum CRP levels (≤10 mg/L) compared to the SC group (*p* = 0.02) (Wanaratna et al., 2022). In Shanker’s 2023 study, a significant reduction in serum CRP was observed within the APE group from baseline to 4 days post-treatment (*p* = 0.01), though a direct comparison with the comparator group was not available (Shanker et al., 2023), leaving the broader relevance of this finding uncertain (Table 2).

#### 3.4.5 Serum IL-6 levels

The trends in serum IL-6 levels were similar to those observed with CRP, with the pooled estimates from two trials (n = 228) (Siripongboonsitti et al., 2023; Prasoppokakorn et al., 2024) showed no significant difference between AP and antivirals at the final follow-up, with a pooled MD of −0.07 (95%CI; −0.17 to 0.03) without heterogeneity (I^2^ = 0.0%) (Figure 3d).

Nevertheless, significant short-term improvements were reported in individual studies. In Prasoppokakorn’s 2024 trial, serum IL-6 levels were significantly lower in the APE group at 7 days compared to the antiviral group (*p* = 0.001), a difference that was not sustained at 14 days (Prasoppokakorn et al., 2024). Furthermore, Shanker’s 2023 study also identified a significant reduction in IL-6 levels within the APE group from baseline to 4 days (*p* = 0.01), but this study similarly lacked comparative data for the comparator group (Shanker et al., 2023), making it challenging to generalize the finding (Table 2).

#### 3.4.6 Adverse outcomes

Adverse events were predominantly mild and transient, with elevated liver enzymes being the most common. Notably, Siripongboonsitti’s study reported mild hepatitis in 24.6% of participants concurrently receiving APE and antivirals, but all cases resolved within 28 days (Siripongboonsitti et al., 2023). No significant differences in adverse event profiles were observed between AP and comparator groups in any of the included studies (Wanaratna et al., 2022; Zhang et al., 2021; Kanokkangsadal et al., 2023; Shanker et al., 2023; Siripongboonsitti et al., 2023; Prasoppokakorn et al., 2024). Overall, AP was generally well-tolerated.

## 4 Discussion

The emergence of SARS-CoV-2 created global uncertainty, with limited prior information on its pathology and treatment, including varied pandemic responses (Wiersinga et al., 2020; World Health organization, 2021; Srisubat et al., 2023; Centers for Disease Control and Prevention, 2024; Greenhalgh et al., 2024; Department of Disease Control, 2023), leading to methodological variability in early clinical trials. Heterogeneous study designs, inconsistent outcome definitions or measures, and differences in follow-up durations weakened the evidence base, underscored the need for harmonized methodologies and standardized measurements to establish a stronger scientific foundation.

AP, widely used in traditional medicine for respiratory and inflammatory conditions, is rich in diverse secondary metabolites, notably diterpenoid lactones (AG, 14-deoxyandrographolide, neoandrographolide, 14-deoxy-11,12-didehydroandrographolide), flavonoids, and phytosterols, which collectively exhibit anti-inflammatory, antiviral, and immunomodulatory properties. Mechanistically, AG and its derivatives modulate inflammatory pathways by suppressing NF-κB, COX-2, iNOS, and proinflammatory cytokines, while enhancing lymphocyte proliferation and interleukin-2 expression. Notably, *in silico* studies demonstrated strong binding affinities of these compounds to SARS-CoV-2 targets, including the spike protein, spike protein-angiotensin-converting enzyme 2 (ACE-2) receptor complex, main protease (Mpro), papain-like protease (PLpro), RNA-dependent RNA polymerase (RdRp), and N-protein RNA-binding domain, supporting their proposed role in COVID-19 management. In clinical contexts, AP has been shown to alleviate symptoms of URTI and demonstrated immunomodulatory benefits in human immunodeficiency virus (HIV)-positive individuals, though its specific efficacy in COVID-19 remains under investigation. These pharmacological properties and mechanistic insights underscore the rationale for evaluating AP as an adjunctive therapy in SARS-CoV-2 infections (Wagner et al., 2015; Hu et al., 2017; Intharuksa et al., 2022; Siridechakorn et al., 2023; Udupa et al., 2025).

The identical clinical and biomarker outcomes between the adjunctive AP and antiviral groups could be attributed to pharmacokinetic limitations of AP-derived compounds, including poor solubility, low oral bioavailability, and extensive hepatic metabolism, resulting in suboptimal plasma concentrations and potentially compromising therapeutic efficacy (Loureiro Damasceno et al., 2022). Four included studies administered APE at 180 mg AG/day, in three divided oral doses, with comparator groups receiving favipiravir (Wanaratna et al., 2022; Kanokkangsadal et al., 2023; Siripongboonsitti et al., 2023; Prasoppokakorn et al., 2024), in alignment with Thailand’s COVID-19 treatment guideline (Department of Disease Control, 2023). This AP dosage recommendation was based on URTI studies with inflammatory components (Songvut et al., 2022). Interestingly, Zhang’s 2021 study demonstrated that direct IV administration of more water-soluble synthetic AG derivatives significantly accelerated cough and overall symptom resolution compared to the SC group (Zhang et al., 2021), highlighting potential advantages of optimized delivery forms, dosage, and administration strategies.

Additionally, another possible explanation for the comparable outcomes between groups was the limited distinction between AP and comparator treatments, as the study sample primarily consisted of non-severe COVID-19 individuals, whose symptoms were further mitigated by favipiravir (Wanaratna et al., 2022; Kanokkangsadal et al., 2023; Shanker et al., 2023; Siripongboonsitti et al., 2023; Prasoppokakorn et al., 2024) or remdesivir (Shanker et al., 2023). Moreover, the extended follow-up period likely facilitated substantial recovery in both groups, potentially obscuring early meaningful differences given the rapid progression and recovery trajectory of COVID-19 (Wiersinga et al., 2020; World Health organization, 2021). Notably, significant reductions in inflammatory markers were observed within 7 days in some studies but diminished by subsequent evaluations as recovery progressed (Zhang et al., 2021; Kanokkangsadal et al., 2023; Shanker et al., 2023; Siripongboonsitti et al., 2023; Prasoppokakorn et al., 2024). For instance, Prasoppokakorn’s 2024 study reported significantly lower serum CRP and IL-6 levels in the APE group at 7 days post-treatment compared to antivirals, which equalized by day 14 (Prasoppokakorn et al., 2024). Additionally, Shanker’s 2023 study, which compared APE with antivirals, further reported a shorter time to overall clinical improvement in the APE group (4.17 ± 0.56 days post-treatment) compared to the comparators (6.23 ± 1.95 days post-treatment), despite using a lower dosage of AG. Although these improvements were evident, the high RoB due to missing outcome data limited the reliability of these findings for clinical decision-making (Shanker et al., 2023). Nevertheless, these differences suggested that AP may exert its effects earlier in the disease course.

These early effects of AP observed in COVID-19 trials are consistent with previous meta-analyses demonstrating its efficacy in reducing inflammation and alleviating symptom severity in URTI (Hu et al., 2017) and viral cough (Wagner et al., 2015). Such findings support the potential role of AP as an adjunctive herbal intervention for respiratory viral infections with inflammatory components. However, the challenges in COVID-19 studies remain unique due to the disease’s rapidly evolving nature, heterogeneity in patient severity, and regional variation in circulating viral strains (Wiersinga et al., 2020; World Health organization, 2021). Notably, only one study confirmed participant-level viral genotyping, reporting Delta and Alpha variants (Siripongboonsitti et al., 2023), while the remaining trials lacked variant-specific data. Although variant inference based on regional epidemiology suggests the likely predominance of the Wuhan/Original strain (Wanaratna et al., 2022; Zhang et al., 2021), Alpha (Wanaratna et al., 2022), Delta, or Omicron (Kanokkangsadal et al., 2023; Prasoppokakorn et al., 2024) in respective studies, this remains speculative. Nevertheless, contextualizing these findings within known variant trends is essential for interpreting clinical outcomes, though the absence of systematic genotyping represents a key limitation.

Despite inconclusive evidence, the observed trends in COVID-19 studies suggested potential clinical benefits of AP in managing viral infections. Given that SARS-CoV-2 itself can cause liver damage independent of treatment, the mild hepatitis observed in some studies may not be directly attributable to AP (Wiersinga et al., 2020; Wanaratna et al., 2022; Zhang et al., 2021; Kanokkangsadal et al., 2023; Shanker et al., 2023; Siripongboonsitti et al., 2023; Prasoppokakorn et al., 2024). With its robust safety profile demonstrated in the included studies (Wanaratna et al., 2022; Kanokkangsadal et al., 2023; Shanker et al., 2023; Siripongboonsitti et al., 2023; Prasoppokakorn et al., 2024) and previous meta-analyses (Wagner et al., 2015; Hu et al., 2017; Worakunphanich et al., 2021), AP emerges as a promising natural adjunctive treatment, particularly for patients seeking complementary antiviral therapies.

Our systematic review and meta-analysis emphasized the importance of investigating *Andrographis peniculata* (Burm. f.) Nees (AP) as a potential natural adjunctive therapy for COVID-19, with resulting trends indicating possible benefits in symptom improvement, fever and cough resolution, and reductions in CRP and IL-6 levels. These findings underscore the need for further research to optimize its therapeutic potential and explore its role in addressing emerging clinical challenges, such as long COVID, that continue to affect millions worldwide. Developing innovative therapeutic strategies incorporating AP could provide valuable insights and support the advancement of evidence-based approaches to improve patient outcomes across different phases of the disease.

### 4.1 Limitations

The findings of this study were limited by methodological variability, including a high risk of bias in several studies, study design heterogeneity, inconsistent outcome definitions, and variations in follow-up durations, all of which impeded comprehensive analysis and result interpretation. Key constraints included small sample sizes, variability in outcome measurements, and overall study designs, which hindered the detection of meaningful differences. The limited number of studies for certain outcomes precluded the generation of funnel plot to assess publication bias, affecting the reliability and generalizability of the findings. Additionally, pooling data for meta-analysis was complicated by divergent outcome measurement methods between studies using antivirals and SC alone comparators. As COVID-19 cases decline globally, maintaining the focus on research with the momentum of large-scale trials becomes challenging. Future research should prioritize improving APE, or AP-derived compounds bioavailability through formulation advancements, or alternative delivery methods, while also employing larger sample sizes, harmonized outcome measures, and more frequent follow-ups to capture early treatment effects. Adopting rigorous and standardized methodologies remains crucial to establish a robust evidence base.

## Registration and protocol

This study was conducted in accordance with the Cochrane guideline for a systematic review of interventions and PRISMA 2020 statement (RRID:SCR_018721) (Page et al., 2021b; Page et al., 2021a; Akl et al., 2024). The protocol was prospectively registered in PROSPERO (RRID:SCR_019061) on November 12, 2024 (CRD42024608858).

## Data Availability

The original contributions presented in the study are included in the article/Supplementary Material, further inquiries can be directed to the corresponding authors.
