# Correction: Exploring the clinical effects of *Andrographis paniculata*-derived compounds, its extract, or derivatives for the treatment of COVID-19: a systematic review and meta-analysis

**DOI:** 10.3389/fphar.2026.1823854

**Published:** 2026-05-22

**Authors:** Peerapong Prabhakornritta, Neti Waranuch, Anjana Fuangchan, Kantapich Srikham, Kansak Boonpattharatthiti, Cindy Barnig, Siwaporn Boonyasuppayakorn, Tasana Pitaksuteepong, Parvapan Bhattarakosol, Brice Moulari, Yann Pellequer, Teerapon Dhippayom

**Affiliations:** 1 Cosmetics and Natural Products Research Center (CosNat), Faculty of Pharmaceutical Sciences, Naresuan University, Phitsanulok, Thailand; 2 Department of Pharmaceutical Technology, Faculty of Pharmaceutical Sciences and Center of Excellence for Innovation in Chemistry, Naresuan University, Phitsanulok, Thailand; 3 Université Marie et Louis Pasteur, EFS, INSERM UMR1098 RIGHT, Besançon, France; 4 The Research Unit of Evidence Synthesis (TRUES), Faculty of Pharmaceutical Sciences, Naresuan University, Phitsanulok, Thailand; 5 Department of Chest Diseases, University Hospital of Besançon, Besançon, France; 6 Center of Excellence in Applied Medical Virology, Department of Microbiology, Faculty of Medicine, Chulalongkorn University, Bangkok, Thailand; 7 Department of Pharmacotherapy, University of Utah College of Pharmacy, Salt Lake City, UT, United States

**Keywords:** andrographis, andrographolide, COVID-19, antivirals, systematic review, meta-analysis

In the published article, there was an error in Table 1 concerning Siripongboonsitti, 2023 (Thailand) and Shanker et al., 2023 (India). The intervention and comparator descriptions were incorrectly presented and may have led to misunderstanding of the treatment arms.

For Siripongboonsitti, 2023, the comparator intervention was displayed as:

“Favipiravir (a loading dose of 3,600 mg on day 0, followed by a daily dose of 1,600 mg from days 1 to 4) with SC.”

It should read:

“Placebo 3 capsules po tid and favipiravir (a loading dose of 3,600 mg on day 0, followed by a daily dose of 1,600 mg from days 1 to 4) with SC.”

For Shanker et al., 2023, the experimental and comparator interventions were displayed as:

“CIM-MEG19 (200 mg providing APE 150 mg/tablet, with analyzed AG 35–40 mg/g), 1 tablet po bid; adjunctive to SC (n = 3) or antivirals (remdesivir (n = 24), favipiravir (n = 13)) (for 4 days)” and “Placebo 1 capsule po od with SC (n = 3) or antivirals (remdesivir (n = 24), favipiravir (n = 13))”, respectively.

They should read:

“CIM-MEG19 (200 mg providing APE 150 mg/tablet, with analyzed AG 35–40 mg/g), 1 tablet po bid; adjunctive to SC (n = 40) (for 4 days)” and “SC (n = 3) or SC with antivirals (remdesivir (n = 24), favipiravir (n = 13))”, respectively.

The corrected Table 1 appears below.

The original article has been updated.

**TABLE 1:** Characteristics of included studies.

Study, year (country)	Setting	Population (n)	Age (y)	Male (%)	Duration (day)	Experimental intervention	Comparator intervention	Outcome measures	Funding support
Prasoppokakorn, 2024 (Thailand) (Prasoppokakorn et al., 2024)	Multicentred, 2 provincial hospitals	Mild-to-moderate COVID-19 (n = 82)	43.9 ± 15.2	53.66	14	APE (60 mg AG/capsule), 1 capsule po tid (providing 180 mg AG/day); adjunctive to Favipiravir and SC (for 5 days)	Favipiravir (a loading dose of 3,600 mg on day 0, followed by a daily dose of 1,600 mg from days 1 to 4) with SC	Fever and cough resolution rates, CRP, IL-6, and adverse outcomes (elevated average liver enzymes without hepatitis)	Academic funding
Siripongboonsitti, 2023 (Thailand) (Siripongboonsitti et al., 2023)	Single centred, 1 hospital	Mild and moderate COVID-19 (n = 146)	EG 35 (26–46) CG 41 (28–51)	43.84	14	APE (20 mg AG/capsule), 3 capsules po tid (providing 180 mg AG/day); adjunctive to Favipiravir and SC (for 5 days)	Placebo 3 capsules po tid and favipiravir (a loading dose of 3,600 mg on day 0, followed by a daily dose of 1,600 mg from days 1 to 4) with SC	WHO-CPS improvement, fever and cough resolution rates, CRP, IL-6, and adverse outcomes (mild and moderate hepatitis)	Academic funding
Shanker, 2023 (India) (Shanker et al., 2023)	Multicentred, 2 hospitals	Mild-to-moderate COVID-19 (n = 80)	41–60	68.75	4	CIM-MEG19 (200 mg providing APE 150 mg/tablet, with analyzed AG 35–40 mg/g), 1 tablet po bid; adjunctive to SC (n = 40) (for 4 days)	SC (n = 3) or SC with antivirals (remdesivir (n = 24), favipiravir (n = 13))	Time to WHO ordinal clinical severity scale (2-point) improvement, hs-CRP, IL-6, negative COVID-19 PCR	Healthcare industry funding
Kanokkangsadal, 2023 (Thailand) (Kanokkangsadal et al., 2023)	Single centred, university field hospital	Mild-to-moderate COVID-19 (n = 165)	EG 29 (23, 35) CG 33 (25, 42)	33.33	5	APE (20 mg AG/capsule), 3 capsules po tid (providing 180 mg AG/day); with SC (for 5 days)	Placebo 3 capsules po tid and SC	WHO-CPS improvement (changing >3), Fever and cough severity (clinical COVID-19 symptoms 0–10 numeric rating scale)	Academic funding
Wanaratna, 2022 (Thailand) (Wanaratna et al., 2022)	Multicentred, 2 state quarantine hospitals	Mild COVID-19 (n = 57)	EG 39.3 ± 11.4 CG 39.4 ± 11.6	40.35	5	APE (20 mg AG/capsule), 3 capsules po tid (providing 180 mg AG/day); with SC (for 5 days)	Placebo 3 capsules po tid and SC	Self-assessed complete clinical recovery from VAS scores, low CRP level (≤10 mg/L), negative COVID-19 RT-PCR	Academic funding
Zhang, 2021 (China) (Zhang et al., 2021)	Multicentred, 5 hospitals	Mild-to-moderate COVID-19 (n = 130)	EG 44.31 ± 13.45 CG 48.25 ± 14.22	46.15	14	Xiyanping (9-dehydro-17-hydroandrographolide, and sodium 9-dehydro-17-hydro-andrographolide-19-yl sulfate) injection (10 mg AG/kg, not exceed 500 mg/day); with SC (for 7–14 days)	SC	Time to complete symptom, fever, and cough resolutions, and time to virus clearance (2 consecutive nucleic acid tests)	Academic funding

Data are reported as mean ± standard deviation (SD) or mean (range) or median (first quartile (Q1), third quartile (Q3)).

EG, experimental group; CG, comparator group; COVID-19, coronavirus disease of 2019; HT, hypertension; DM, diabetes; DLP, dyslipidemia; CVD, cardiovascular diseases; CKD, chronic kidney disease; EG, experimental group; CG, comparator group; APE, *Andrographis paniculata* (Burm. f.) nees extract; AG, andrographolide; SC, supportive care; WHO-CPS, COVID-19, World Health Organisation clinical progression scale; VAS, visual analogue scale; CRP, C-reactive protein; hs-CRP, high-sensitivity CRP; IL, interleukin; RT-PCR, real-time polymerase chain reaction; po, per os (oral administration); tid, ter in die (three times a day); od, omne in die (once a day).

