# Supplementary material for: Exploring the clinical effects of Andrographis paniculata-derived compounds, its extract, or derivatives for the treatment of COVID-19: a systematic review and meta-analysis
Source: Front Pharmacol. 2025 Jul 31;16:1598255. doi: 10.3389/fphar.2025.1598255 (PMC12351123; doi:10.3389/fphar.2025.1598255)
Supplement: Supplementary file 1 [file Table1.docx]

# Supplementary Material

Table S1. Search strategy

| **Database: Cochrane Central Register of Controlled Trials (CENTRAL) (RRID:SCR_006576)** | | |
| --- | --- | --- |
| **Search number** | **Query** | **Results** |
| #1 | (COVID):ti,ab,kw | 21,921 |
| #2 | (SARS-CoV-2) ):ti,ab,kw | 586 |
| #3 | MeSH descriptor: [COVID-19] explode all trees | 8,217 |
| #4 | MeSH descriptor: [SARS-CoV-2] explode all trees | 3,484 |
| #5 | (androgr*):ti,ab,kw | 155 |
| #6 | MeSH descriptor: [Andrographis] explode all trees | 18 |
| #7 | (antivir*):ti,ab,kw | 14,554 |
| #8 | (iverm*):ti,ab,kw | 1,111 |
| #9 | (favi*):ti,ab,kw | 584 |
| #10 | (molnu*):ti,ab,kw | 117 |
| #11 | (remde*):ti,ab,kw | 510 |
| #12 | MeSH descriptor: [Antiviral Agents] explode all trees | 12,277 |
| #13 | MeSH descriptor: [Ivermectin] explode all trees | 629 |
| #14 | OR/1−4 | 21,969 |
| #15 | OR/5−6 | 155 |
| #16 | OR/7−13 | 21,945 |
| #17 | AND/14−16 | 11 |
| **Database: MEDLINE (RRID:SCR_002185) (through PubMed (RRID:SCR_004846))** | | |
| **Search number** | **Query** | **Results** |
| #1 | COVID[Title/Abstract] | 441,442 |
| #2 | SARS-CoV-2[Title/Abstract] | 237,912 |
| #3 | COVID[MeSH] | 276,162 |
| #4 | SARS-CoV-2[MeSH] | 179,867 |
| #5 | androgr*[Title/Abstract] | 2,229 |
| #6 | Andrographis[MeSH] | 620 |
| #7 | Andrographolide[Supplementary Concept] | 995 |
| #8 | antivir*[Title/Abstract] | 200,272 |
| #9 | iverm*[Title/Abstract] | 10,836 |
| #10 | favi*[Title/Abstract] | 7,047 |
| #11 | molnu*[Title/Abstract] | 884 |
| #12 | remde*[Title/Abstract] | 4,528 |
| #13 | Antiviral Agents[MeSH] | 181,599 |
| #14 | ivermectin[MeSH] | 7,944 |
| #15 | favipiravir[Supplementary Concept] | 672 |
| #16 | molnupiravir[Supplementary Concept] | 345 |
| #17 | remdesivir[Supplementary Concept] | 1,369 |
| #18 | OR/1−4 | 441,442 |
| #19 | OR/5−7 | 2,229 |
| #20 | OR/8−17 | 288,293 |
| #21 | AND/18−20 | 66 |
| **Database: EMBASE (RRID:SCR_001650)** | | |
| **Search number** | **Query** | **Results** |
| #1 | ‘covid’/exp OR covid | 510,989 |
| #2 | ‘sars cov 2’ | 188,410 |
| #3 | ‘coronavirus disease 2019’/exp | 421,956 |
| #4 | androgr* | 4,786 |
| #5 | ‘andrographis’/exp | 2,078 |
| #6 | ‘andrographolide’/exp | 2,677 |
| #7 | antivir* | 293,341 |
| #8 | iverm* | 18,713 |
| #9 | favi* | 13,349 |
| #10 | molnu* | 1,978 |
| #11 | remde* | 14,889 |
| #12 | ‘antivirus agent’/exp | 1,611,282 |
| #13 | ‘ivermectin’/exp | 17,661 |
| #14 | ‘favipiravir’/exp | 5,231 |
| #15 | ‘molnupiravir’/exp | 1,852 |
| #16 | ‘remdesivir’/exp | 14,017 |
| #17 | OR/1−3 | 530,839 |
| #18 | OR/4−6 | 4,786 |
| #19 | OR/7−16 | 1,717,265 |
| #20 | AND/17−19 | 186 |
| **Database: EBSCO Open Dissertations** | | |
| **Search number** | **Query** | **Results** |
| #1 | COVID | 5,264 |
| #2 | sars-cov-2 | 795 |
| #3 | androgr* | 15 |
| #4 | antivir* | 1,857 |
| #5 | iverm* | 130 |
| #6 | favi* | 62 |
| #7 | molnu* | 7 |
| #8 | remde* | 19 |
| #9 | OR/1−2 | 5,585 |
| #10 | OR/4−8 | 2,057 |
| #11 | AND/17-19 | 0 |

COVID-19, coronavirus disease of 2019; SARS-CoV-2, severe acute respiratory syndrome coronavirus 2; MeSH, medical subject headings

Table S2. List of excluded studies

| **Number** | **Reference** | **Summary comment for exclusion** |
| --- | --- | --- |
| #1 | Narayanababu R, Kannan SR, Lenin R, Sakthibalan M, Sudha KM, Gowtham K, Paul A, David E. Evaluation of Efficacy and Safety of Clevira as an Add on Drug in Mild to Moderate COVID-19 Positive PatientsA Randomized Control Trial. JOURNAL OF CLINICAL AND DIAGNOSTIC RESEARCH. 2022 Feb 1;16(2):KC01-7. | Non-single herbal product for experimental intervention |
| #2 | Ratiani L, Pachkoria E, Mamageishvili N, Shengelia R, Hovhannisyan A, Panossian A. Efficacy of Kan Jang^®^ in patients with mild COVID-19: a randomized, quadruple-blind, placebo-controlled trial. Pharmaceuticals. 2023 Aug 22;16(9):1196. | Non-single herbal product for experimental intervention |

COVID-19, coronavirus disease of 2019
